# Microtubule array reorientation in response to hormones does not involve changes in microtubule nucleation modes at the periclinal cell surface

**DOI:** 10.1093/jxb/eru325

**Published:** 2014-08-18

**Authors:** Samantha Atkinson, Angela Kirik, Viktor Kirik

**Affiliations:** School of Biological Sciences, Illinois State University, Normal, IL 61790, USA

**Keywords:** γ-tubulin complex, microtubule cytoskeleton, microtubule organization, microtubule nucleation.

## Abstract

Aligned microtubule arrays spatially organize cell division, trafficking, and determine the direction of cell expansion in plant cells. In response to changes in environmental and developmental signals, cells reorganize their microtubule arrays into new configurations. Here, we tested the role of microtubule nucleation during hormone-induced microtubule array reorientation. We have found that in the process of microtubule array reorientation the ratios between branching, parallel, and *de-novo* nucleations remained constant, suggesting that the microtubule reorientation mechanism does not involve changes in nucleation modes. In the *ton2/fass* mutant, which has reduced microtubule branching nucleation frequency and decreased nucleation activity of the γ-tubulin complexes, microtubule arrays were able to reorient. Presented data suggest that reorientation of microtubules into transverse arrays in response to hormones does not involve changes in microtubule nucleation at the periclinal cell surface

## Summary

Microtubules are oriented into transverse arrays in response to growth-promoting hormones. This reorientation process does not involve changes in the proportion of different nucleation modes at the periclinal cell surface.

## Introduction

Cells arrange microtubule polymers into different types of co-aligned arrays. Microtubule orientation is instrumental in chromosome separation, cell division, and for directionality of trafficking processes in polarized cells. In growing interphase plant cells the pattern of cortical microtubules directs cell expansion by guiding deposition of cellulose microfibres in the cell wall (Paredez *et al.*, 2006; Chan *et al.*, 2011). Orientation of cortical microtubules depends on developmental and environmental signals. Microtubules are aligned perpendicular to the direction of cell elongation in rapidly elongating hypocotyl cells, and switch to a longitudinal orientation when cell growth becomes slower (Sambade *et al.*, 2012). Light exposure suppresses cell elongation and promotes the transition from transverse to longitudinal microtubule arrays in dark-grown seedlings (Kigel and Cosgrove, 1991; Paredez *et al.*, 2006). Conversely, growth-promoting hormones auxin, gibberellic acid, and brassinosteroids drive the formation of transverse microtubule arrays (Shibaoka, 1993; Duckett and Lloyd, 1994; Fujino *et al.*,1995; Sambade *et al.*, 2012; Wang *et al.*, 2012; Vineyard *et al.*, 2013).

The process of cortical microtubule rearrangement is not well understood. Recent findings show that during microtubule reorientation several mechanisms need to be coordinated, including the formation of new microtubules in the discordant orientation, their amplification, and interactions with other microtubules. Generally, reorientation begins with the appearance of discordant microtubules that effectively contribute to new arrays (Yuan *et al.*, 1995; Sambade *et al.*, 2012; Lindeboom *et al.*, 2013; Vineyard *et al.*, 2013). In the longitudinal to transverse reorientation stimulated by rapid cell elongation, transverse microtubules appear from the midzone of orthogonal sidewalls, forming an isotropic transition array in the star or basket-like configuration at the onset of reorientation (Sambade *et al.*, 2012; Vineyard *et al.*, 2013). As cells enter the rapid elongation phase, more transverse microtubules appear from the longitudinal sidewalls, spreading as a wave towards cell ends (Sambade *et al.*, 2012). The appearance of transverse discordant microtubules is stimulated by cell growth promoting hormones gibberellic acid and auxin, whereas auxin hormone inhibitors suppress transverse microtubules (Sambade *et al.*, 2012; Vineyard *et al.*, 2013).

Discordant microtubules were shown to amplify selectively through a microtubule severing mechanism (Lindeboom *et al.*, 2013). In the process of light-induced reorientation from transverse to longitudinal arrays, growing ends of seeding longitudinal microtubules encounter pre-existing transverse microtubules, and often form crossovers. Microtubule crossovers attract katanin proteins that sever new microtubules preferentially, creating new plus ends that can either start depolymerization or, alternatively, can initiate a new growing end (Wightman *et al.*, 2007; Lindeboom *et al.*, 2013; Wightman *et al.*, 2013). The new growing ends can initiate more plus ends, leading to a rapid amplification of longitudinally oriented microtubules.

Selective removal of longitudinally oriented microtubules has been suggested to lead to a transverse orientation of microtubule arrays (Ehrhardt and Shaw, 2006). A microtubule-associated protein, CLASP, has been shown to accumulate and suppress catastrophes at the borders between periclinal and anticlinal cell surfaces (Ambrose *et al.*, 2011). Such localized anticatastrophy activity driven by CLASP localized along the longitudinal sidewalls promotes transverse ordering of microtubules by preferential removal of longitudinal microtubules (Ambrose *et al.*, 2011). Time-lapse analysis of reorganizing microtubule arrays after treatment with a gibberellic acid and auxin hormone mix showed an increase in the frequency of transverse microtubules during array reorientation, suggesting an active mechanism promoting formation of new microtubules in the transverse orientation (Sambade *et al.*, 2012; Vineyard *et al.*, 2013). Furthermore, hormone induction rapidly depleted the number of plus ends on the periclinal surface (Vineyard *et al.*, 2013), and nucleation of new microtubules was decreased during light-induced microtubule reorientation (Lindeboom *et al.*, 2013), suggesting that the mechanism of microtubule array reorientation involves changes in new microtubule initiation.

Mechanisms generating discordant microtubules at the beginning of the reorientation process are unknown. Nucleation of new microtubules by γ-tubulin complexes is instrumental in formation of oriented microtubules in spindles and phragmoplasts (Hotta *et al.*, 2012; Murata *et al.*, 2013; Petry *et al.*, 2013). In the process of branching nucleation at the γ-tubulin complexes, new microtubules are created at 30–60º angles toward extant microtubules (Murata *et al.*, 2005; Chan *et al.*, 2009; Nakamura and Hashimoto, 2009). Thus, branching nucleations are in position to initiate transverse microtubules by two subsequent branching nucleation events or by a branching nucleation followed by a deflection of the new microtubule path towards a steeper angle (Lindeboom *et al.*, 2013). Mutations in *TON2* and *AUG8* genes that affect microtubule branching nucleation were shown to have microtubule reorientation defects (Kirik *et al.*, 2012; Cao *et al.*, 2013). However, in addition to a reduction in microtubule branching nucleation, both mutations affect microtubule stability making the connection between microtubule branching nucleation mode and reorientation less clear.

In this study we tested directly the relationship between microtubule nucleation and reorientation of microtubule arrays induced by hormones. Microtubule nucleations were analysed using long time-lapse imaging of the reorganizing microtubule arrays with GCP2–GFP labelled γ-tubulin nucleation complexes. Using the *ton2* mutant, which affects microtubule branching nucleation, we tested the role of branching nucleation during reorientation from longitudinal to transverse arrays.

## Material and methods

### Plant growth conditions

*Arabidopsis thaliana* plants were grown in light cabinets at 25 °C with a 16h day and 8h night light period. Columbia-0 ecotype was used as the wild type in this study. Seeds were germinated on half-strength Murashige and Skoog (MS) media with no sugar and grown for 4–5 days before imaging. Plant lines carrying the mCherry:TUB5/GCP2:GFP and YFP:TUA5 microtubule markers were created in previous studies (DeBolt *et al.*, 2007; Nakamura *et al.*, 2010; Kirik *et al.*, 2012).

### Hormone treatments

For observation of microtubule reorientation over a 2h time period, we used the combination of gibberellic acid (GA_4_; Sigma) and auxin (IAA; Sigma) hormones at concentrations described in the previous microtubule reorientation study (Vineyard *et al.*, 2013). For 90min time-lapse analysis, seedlings were mounted in 70 µl of 20 µM GA_4_/ 1 µM IAA hormone mix solution in the half-strength MS media. In control experiments we used the same concentration of solvents (DMSO and ethanol) as in the hormone-treatment experiments. Silicon vacuum grease (Dow Corning) was used to seal coverslips to prevent evaporation during imaging.

### Microscopy and data analysis

Live-cell imaging was performed on a spinning-disk confocal microscope with a Leica DM6000 inverted microscope, Yokogawa CSUX scanner, and Photometrics Evolve 512 camera. YFP/GFP and Cherry were excited at 488nm and 561nm, respectively. The time-lapse imaging was set up with 6 s intervals for a total of 900 time points (1.5h), using the ×100 oil immersion objective. Images were analysed using ImageJ (W. Rasband, National Institutes of Health, Bethesda, MD) and Photoshop (Adobe Systems).

For observation of microtubule reorientation over a 2h time period, microtubule arrays were visualized with the yellow fluorescent protein fused to alpha-tubulin5 (YFP:TUA5). Images of the hypocotyl cells were taken every 30min.

GCP2:GFP-labelled microtubule nucleation complexes that were stabilized at the cell cortex for at least 6 s were tested for branching, *de novo*, parallel, or failed nucleations. Failed nucleations were defined as events when nucleation complexes were stable for at least two frames (6 s) but did not initiate nucleation.

Microtubules coming from longitudinal cell sides were counted in plants expressing the YFP:TUA5 microtubule marker and the numbers were normalized by dividing them by the length of the side walls. Microtubules emerging from the sides were counted as transverse if their angle to the longitudinal cell axis was in the range of 65–115° to the longitudinal cell axis. All images where background-subtracted with the Rolling Ball algorithm (ImageJ).

## Results

### Reorientation of microtubule arrays in response to auxin/gibberellic acid treatment is attenuated in the *ton2* mutants

Mutation in the *Arabidopsis* PP2A B’’ regulatory subunit FASS/TON2 leads to changes in microtubule nucleation modes such that the proportion of branching nucleation is significantly decreased, whereas the proportions of parallel and *de-novo* nucleation modes are slightly higher in mutant cotyledon cells (Kirik *et al.*, 2012). Transversely oriented microtubule arrays in the *ton2* mutant are not able to reorient efficiently into the longitudinal configuration in response to light. Using treatment of light-grown seedlings with a combination of the hormones gibberellic acid (GA_4_) and indole-3-acetic acid (IAA) (Vineyard *et al.*, 2013), we tested here whether *TON2* function is required in longitudinal to transverse microtubule reorientation.

**Fig. 1.**
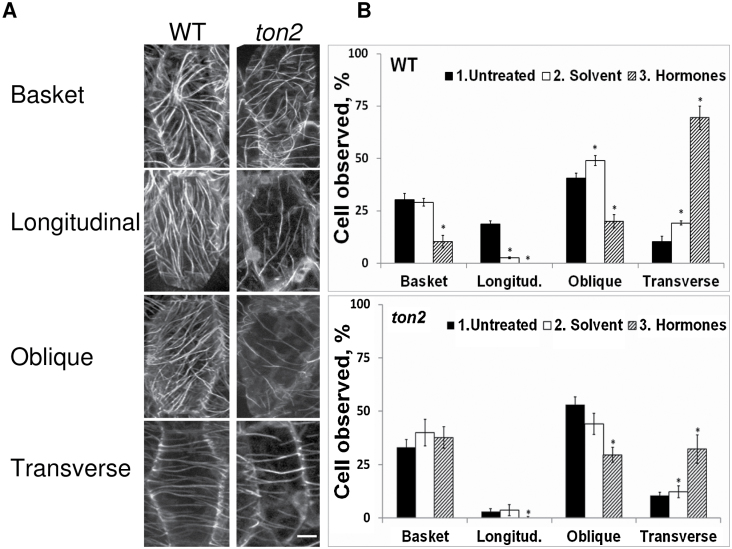
Cortical microtubule array reorganization in *ton2* epidermal hypocotyl cells under treatment with hormones. (A) Different types of microtubule array organization in 4-day-old wild type and *ton2* mutant cells. (B) Configurations of microtubule arrays before treatment (Untreated), after 2h treatment with a control solution containing hormone solvent (Solvent), and after 2h of GA_4_/IAA treatment (Hormones). Bar=10 µm. 30 cells from 6 different plants were analysed. *, *P*<0.05 for differences with untreated plants. The Angle Tool from ImageJ was used to measure orientations of microtubule bundles relative to the longitudinal cell axis. Microtubule arrays with more than 75% of microtubule bundles oriented at 0–19° were classified as longitudinal, microtubules oriented at 20–64° were classified as oblique, and microtubules oriented at 65–115° were classified as transverse.

Light-grown 5 day-old epidermal hypocotyl cells of the *ton2* mutant showed all microtubule array configurations previously described for wild type: basket, longitudinal, oblique, and transverse (Vineyard *et al.*, 2013; Fig. 1A). However, the relative proportions of different microtubule arrays were different in the *ton2* mutant (Fig. 1B). The fraction of cells with longitudinal microtubule arrays was 3.0±1.2% in the *ton2* mutant compared with 18.7±1.6% in wild type. The fraction of oblique microtubule-arrays was 53.0±3.7% in the mutant and 40.5±2.4% in wild type. The fractions of basket and transverse arrays were similar (Fig. 1B).

Treatment with the GA_4_/IAA mix changed the proportion of different microtubule configurations both in wild type and in the *ton2* mutant, but reorientation of microtubules to transverse arrays was attenuated in the mutant. After 2h of hormone treatment, 69% of microtubule arrays became transversely oriented in wild type, compared with 32% in the *ton2* mutant (Fig. 1B). This corresponds to a 7-fold increase in the fraction of transverse arrays in wild type and a 3-fold increase in the *ton2* mutant. In control experiments with solvents used at the same concentration as in the hormone solution, the fraction of transverse arrays increased 1.8-fold in wild type and 1.2-fold in the *ton2* mutant, indicating that the hormone treatment was effective.

Most initial array configurations responded similarly to the treatment. All longitudinal microtubule-arrays were reoriented in wild-type hypocotyl cells, and this array configuration disappeared in hormone-treated cells (Fig. 1B, upper graph). Similarly, the fraction of longitudinal microtubule arrays decreased 10 times in the *ton2* mutant (Fig. 1B, lower graph). Oblique microtubule arrays decreased significantly in wild type and the *ton2* mutant (from 40.6±4.9% to 20.1±3.5 % in wild type and from 53.0±3.7% to 29.6±3.5% in the *ton2* mutant). In contrast, the fraction of hypocotyl cells with a basket configuration dropped from 30.3±2.9% to 10.4±3.0% in wild type and did not change in the *ton2* mutant. After the 2h treatment the fraction of cells with the transverse configuration was significantly smaller and the fractions of basket and oblique cells were higher in the *ton2* mutant (*P*<0.05 *t*-test). These results indicate that TON2 function is required, directly or indirectly, for efficient hormone-induced microtubule reorientation.

**Fig. 2.**
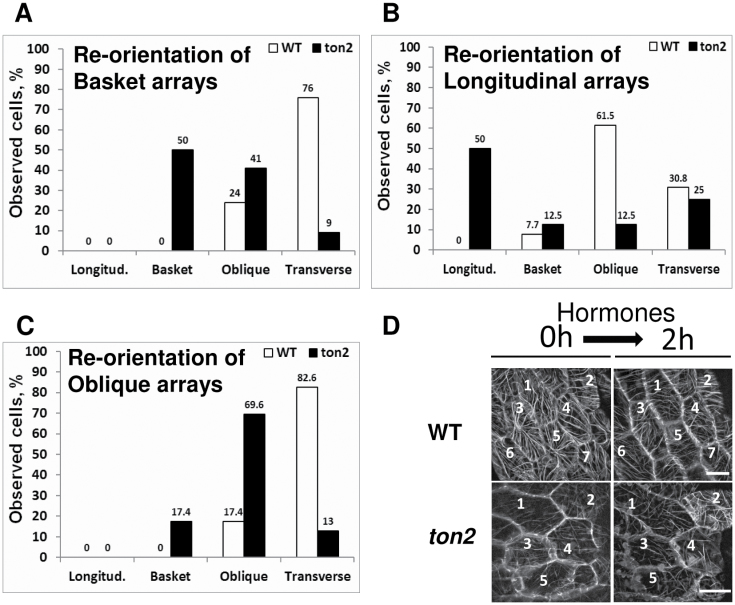
Microtubule array orientation in wild-type and *ton2* hypocotyl cells after 2h of hormone treatment starting from different configurations. (A) All (100%) of basket arrays in wild type (WT) and 50% of basket arrays in the *ton2* mutant were reoriented during 2h hormone treatment into oblique and transverse microtubule arrays. Half of the *ton2* basket arrays did not reorient. No basket arrays became longitudinal. (B) Longitudinal arrays of wild type and *ton2* reoriented (100% in wild type and 50% in the *ton2* mutant) into basket, oblique and transverse configurations; 50% of *ton2* longitudinal microtubule arrays did not reorient. (C) Oblique arrays were reoriented into basket (in the *ton2* mutant only) or transverse configurations; 17% of wild type and 69.6% of *ton2* arrays did not reorient. (D) Examples of microtubule array configurations before and after hormone treatment. Microtubule configurations of individual cells were recorded before and after 2h of hormone treatment. Bar=10 µm; 5 different plants were analysed with 17 wild type-cell and 22 *ton2* cells in initial basket configuration, 13 wild-type and 8 *ton2* cells in initial longitudinal configuration, 23 wild-type and 23 *ton2* cells in initial oblique configuration.

To test reorientation of microtubule arrays in different initial configurations we tracked individual cells over 2h of treatment using a spinning disc confocal microscope (Fig. 2D). All cells with basket arrays (*n*=17 cells from 5 different plants) were reoriented into oblique (24%) and transverse (76%) arrays in wild type (Fig. 2A). The transition from basket to a longitudinal array was not observed, in agreement with observed directionality of hormone-induced transitions from longitudinal to basket, to oblique and finally to transverse configuration (Vineyard *et al.*, 2013). In the *ton2* mutant (*n*=22 cells from 5 plants), 50% of cells with basket arrays did not change their configuration, and the majority of reoriented arrays became oblique (41%) and only 9% became transverse (Fig. 2A).

In wild type all cells with initially longitudinal microtubule arrays (*n*=13 cells from 5 plants) reoriented so that 7.7% of cells were in basket, 61.5% in oblique, and 30.8% were in transverse configuration (Fig. 2B). In the *ton2* mutant 50% of cells (*n*=8 cells from 5 plants) with longitudinal arrays failed to reorient, 12.5% became basket, 12.5% oblique, and 25% became transverse (Fig. 2B).

Starting from oblique microtubule arrays (*n*=23 cells, 5 different plants), the majority of wild-type arrays (82.6%) were reorganized into transverse arrays and a small fraction (17.4%) did not reorient. In the *ton2* mutant (*n*=23, 5 different plants) the majority (69.6%) of cells with oblique arrays did not reorient and only 13% attained the transverse orientation (Fig. 2C). Of the oblique microtubule arrays in the *ton2* mutant, 17.4% reorganized into basket, a transition not observed in wild type (Fig. 2; Vineyard *et al.*, 2013).

Taken together, as in wild type, microtubule arrays in the *ton2* mutant cells responded to treatment with the GA_4_/IAA hormones by reorientation into transverse arrays. However, a large fraction of microtubule arrays did not reorient and the directionality of microtubule array transformations were less robust, showing “reverse order” transitions.

### Microtubule arrays reorient slower in the *ton2* mutant

To test the rates of microtubule array reorientation in wild type and in the *ton2* mutant, we measured the fraction of cells that reorganized into the transverse array configuration after 30, 60, 90, and 120min of hormone treatment.

**Fig. 3.**
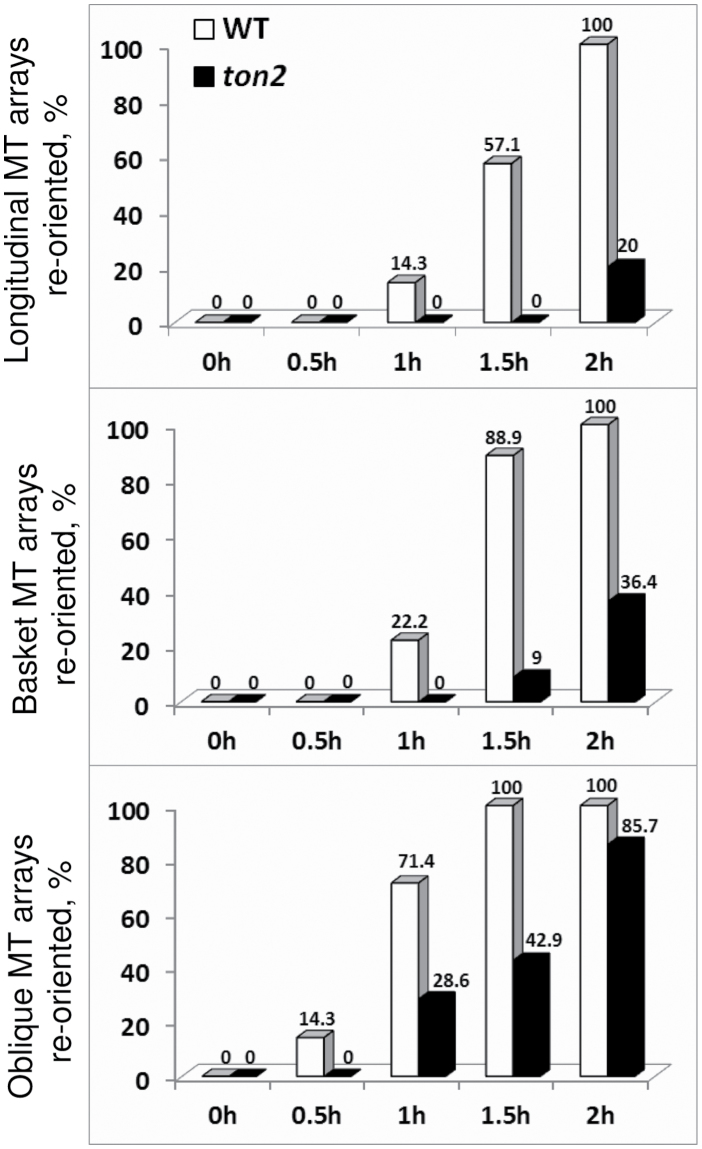
Reorientation into transverse microtubule arrays starting from different microtubule configurations. Bars show the percentage of cell with longitudinal (upper), basket (middle graph) and oblique (lower) microtubule arrays reoriented into transverse orientation. For each configuration 6 cells from 2 different plants were analysed.

Starting from the longitudinal array configuration, transversely oriented arrays appeared in wild type cells after 60min of hormone treatment (14.3%). After 90min 57.1% of cells became transverse and after 120 all cells (*n*=7) transitioned to the transverse configuration. In the *ton2* mutant (*n*=5), no cells with transverse arrays were detected for at least 90min, and after 120min only 20% of cells showed the transverse array configuration (Fig. 3, upper graph).

Cells with the basket array configuration reorganized faster than cells with longitudinal arrays both for wild type and the *ton2* mutant (*n*=9 for wild type and *n*=11 for *ton2*). Compared with wild type, *ton2* mutant cells reoriented slower. After 60min 22.2% of wild-type cells had transverse microtubule arrays, whereas after 90min 88.9% were in a transverse orientation. In the *ton2* mutant only 9% of cells reoriented into transverse arrays after 90min and 36.4% reoriented after 120min (Fig. 3, middle graph).

Both in wild-type and in the *ton2* mutant, cells with oblique arrays (*n*=7 for both wild type and *ton2*) were the fastest to reorganize into transverse arrays, with the first transverse arrays appearing after 30min in wild type and after 60min in the *ton2* mutant (Fig. 3, lower graph).

In summary, cells with longitudinal arrays were the slowest to reorient into transverse arrays, cells with oblique arrays were the fastest, and cells with basket arrays were intermediate. In all cases, *ton2* mutant cells reorganized their arrays slower than wild type.

### Microtubule nucleation modes, efficiency, and branching angles are not changed during hormone-induced microtubule reorientation

Recent data suggested that microtubule branching nucleation may play a role in light-induced reorientation of microtubule arrays from transverse to longitudinal arrays by generating discordant microtubules at the beginning of the reorientation process (Kirik *et al.*, 2012; Cao *et al.*, 2013; Lindeboom *et al.*, 2013). Here we analysed different microtubule nucleation modes during the hormone-induced microtubule reorientation into transverse arrays.

To visualize microtubule nucleation events during the hormone-induced microtubule reorientation we used plants coexpressing GCP2:GFP (nucleation complex marker) and mCherry:TUB5 (microtubule marker) constructs. Time-lapse images of reorienting microtubule arrays were acquired over 90min intervals, in which the majority of wild type cells and approximately one quarter of the *ton2* cells adopted a transverse array configuration.

**Fig. 4.**
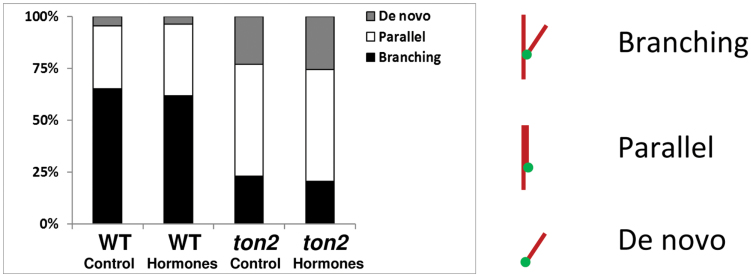
Proportions of branching, parallel, or *de-novo* nucleations in hormone-treated cells. Nucleation data were collected over 90min following the hormone or mock treatment from 4 wild-type cells (from 4 different seedlings) and 3 *ton2* cells (from 3 different seedlings) for each treatments. Branching nucleations comprised 65.2% in solvent-treated wild type, 61.9% in hormone-treated wild type, 23.0% in t*on2* solvent-treated, and 20.5% in *ton2* hormone-treated. Parallel nucleations comprise 30.4% in solvent-treated wild type, 34.36% in hormone-treated wild type, 54.0% in *ton2* solvent-treated, and 53.9% in *ton2* hormone-treated. *De-novo* nucleations comprise 4.4% in solvent-treated wild type, 3.7% in hormone-treated wild type, 23.0% in *ton2* solvent-treated, and 25.65% in *ton2* hormone-treated. (This figure is available in colour at *JXB* online.)

We found that the proportion of different microtubule nucleation types, including branching, nucleations parallel to existing microtubules, and *de-novo* nucleations initiated at the cell cortex areas not occupied by microtubules did not change significantly in wild-type cells treated with hormones (Fishers exact test *P*>0.05; Fig. 4). There was also no effect of hormones on the balance between different nucleation types in the *ton2* mutant. Similar to the reduction of microtubule nucleation frequency during light-induced microtubule reorientation into longitudinal arrays (Lindeboom *et al.*, 2013), the frequency of microtubule nucleation events declined during the hormone-induced transition to transverse arrays (Supplementary Fig. S1, at *JXB* online). To test if exposure to laser light affected microtubule nucleation, we used imaging conditions with greatly reduced acquisition times. Time-lapse images were acquired in the first 5min followed by another 5min acquisition interval after 1h of hormone treatment. We found no change in the ratio of different nucleation modes (Supplementary Fig. S2, at *JXB* online), suggesting that long time-lapse imaging conditions did not affect microtubule nucleation modes.

**Fig. 5.**
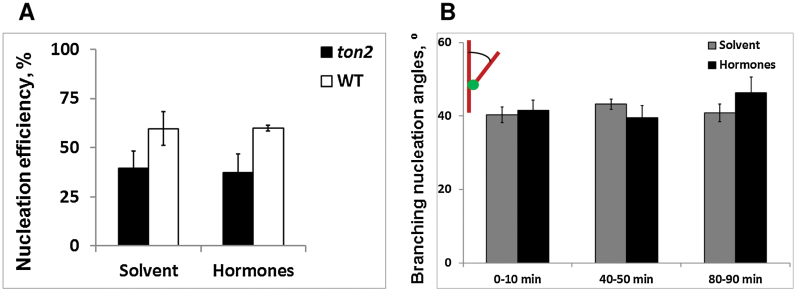
Nucleation efficiency and branching nucleation angles in hormone-treated cells. (A) MT nucleation efficiency from the recruited to the cell cortex γ-tubulin complexes. Nucleation efficiency calculated as a number of total nucleations (branching + parallel + *de novo*) divided by the sum of total nucleations and failed nucleation events. Failed nucleation events were scored as GCP2 complexes which did not promote nucleation during at least 6 s. *n*=4 cells per each case. (B) Branching nucleation angles. In both cases—without hormones and under hormones treatment—average branching nucleation angle stayed around 40°. The distribution of branching nucleation angles did not change significantly during the hormone induced microtubule reorientation (*P*>0.05, two-tailed Student’s T-test). For each time window 4 cells were analysed. Error bars show the standard deviation of the mean (SEM). (This figure is available in colour at *JXB* online.)

To test if hormones influenced the probability to initiate formation of new microtubules from recruited γ-tubulin complexes, we measured the fraction of microtubule initiation events from GCP2–GFP-labelled nucleation complexes stabilized at the cell cortex for at least 6 s. Hormones had no significant effect on the nucleation probability in wild type (59.6% in control, 60% in hormone-treated cells) and in the *ton2* mutant (39.5% in control, 37.5% in hormone-treated cells; Fig. 5A), but the probability to initiate microtubules from stabilized nucleation complexes was significantly reduced in the *ton2* mutant (*P*<0.05, Fisher’s exact test).

In microtubule branching nucleation, the angles of new microtubules vary between 30 and 60°. As microtubule branching at smaller angles lead to increased coalignment (Nakamura and Hashimoto, 2009) and, conversely, microtubule interactions at larger angles were shown to contribute to microtubule array reorientation (Lindeboom *et al.*, 2013), we tested if there was a change in wild type branching nucleation angles during the hormone-induced microtubule reorientation. We found that microtubule branching angles remained constant (Fig. 5B), suggesting that the mechanism of reorientation into a transverse array does not include regulation of the microtubule branching nucleation angles.

### The *ton2* mutant initiates less transversely oriented MTs from the cell sides

Experimental data and computer modelling suggested that transverse microtubules originating at the anticlinal cell sides could drive reorientation of the periclinal microtubule arrays (Sambade *et al.*, 2012; Vineyard *et al.*, 2013). The mechanism generating these transverse microtubules is not known, but it has been speculated that microtubule nucleation and/or selective stabilization of microtubules at the anticlinal side walls may be involved (Ambrose *et al.*, 2011; Sambade *et al.*, 2012; Vineyard *et al.*, 2013). Mutants in the *TON2* gene show reduced microtubule branching nucleation frequency, reduced microtubule stability (Kirik *et al.*, 2012), and a reduced ability of the γ-tubulin complexes to initiate microtubules in hypocotyl cells (Fig. 5A), providing a possibility to test if these microtubule processes play a role in the formation of transversely oriented microtubules originated at anticlinal cell sides.

**Fig. 6.**
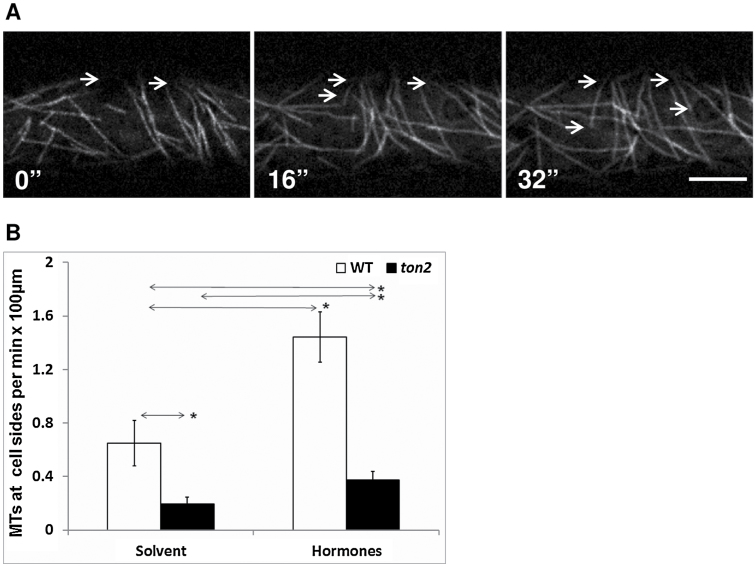
Quantification of microtubules coming from the sides of cells’ midzones during reorientation. (A) Confocal microscope images show new transverse microtubules appearing from the side of the cell midzone (arrows). Bar=10 µm. (B) Frequency of microtubules appearing from cell sides in 2h. For each treatment 4 cells were analysed. Microtubules emerging from the cell sides were counted as transverse if their angels to the longitudinal cell axis were in the 65–115° range. Error bars show the standard deviation of the mean (SEM); *, *P*<0.05.

We tracked the appearance of microtubules from the anticlinal cell sides during 90min of hormone treatment in wild type and in the *ton2* mutant, using a 65–115° angle to the longitudinal cell axis as the criteria for defining these microtubules (Fig. 6A). In the mock-treated cells, the frequency of these emerging transverse microtubules was 3.27-times higher in wild type than in the *ton2* mutant, with 0.7±0.2 microtubules emerging per 1min along the 100 µm of cell side length in wild type and 0.2±0.1 emerging in the *ton2* mutant (Fig. 6B, *P*<0.05). Hormone treatment resulted in a similar increase in frequency of transverse microtubules appearing from the cell sides in wild type and in the *ton2* mutant: wild-type cells showed 2.2-fold increase (1.6±0.2 microtubule emerging in 1min per 100 µm side length) and the *ton2* mutant showed 1.9-fold increase (0.4±0.1 microtubule emerging in 1min per 100 µm side length) (Fig. 6B). Thus, IAA/GA_4_ hormone treatment induced the emergence of transverse microtubules from anticlinal cell sides both in wild type and in the *ton2* mutant, but the number of these microtubules was significantly smaller in the mutant.

## Discussion

In plant cells, most microtubules nucleating from γ-tubulin complexes form branches at an average angle of 40° to existing microtubules. Computer modelling and microtubule organization in mutants with affected branching nucleation angles suggest that branching nucleation promotes dispersion of microtubule arrays and may affect microtubule alignment into parallel arrays (Nakamura and Hashimoto, 2009; Fishel and Dixit, 2013). In the *ton2* mutant, which shows reduced branching nucleation activity, transverse microtubule arrays failed to reorient into longitudinal arrays (Kirik *et al.*, 2012), suggesting a possible causal relationship between branching nucleation and microtubule array reorientation. Here, we tested the role of branching nucleation during hormone-induced microtubule reorientation into transverse arrays using long time-lapse imaging of microtubule nucleation events in wild type and in the *ton2* mutant. We have not found any changes in nucleation modes during the microtubule reorientation process. Similarly, a recent report on light-induced microtubule reorientation also did not provide a strong support for the role of the microtubule branching nucleation in reorientation of microtubule arrays from transverse to longitudinal arrays (Lindeboom *et al.*, 2013). Although a branching nucleation event forming a microtubule in a new orientation was observed during the light-induced reorientation, the rate of microtubule branching nucleation actually decreased in the process of reorientation, suggesting that the reorientation mechanism is not driven by up-regulation of microtubule branching nucleation.

Microtubule arrays in the *ton2* mutant formed the same configurations as wild type and responded to IAA/GA_4_ hormone treatment by orienting transversely to the long cell axis. The reorganization process was less effective in the mutant owing to a combination of a slower reorientation rate and an inability of arrays to reorient in some cells. Less efficient microtubule reorientation in the *ton2* mutant may be caused by reduced sensitivity or response to hormones. However, the number of transverse microtubules appearing from cell sides increased about 3-fold both in wild type and in the *ton2* mutant, suggesting that hormone sensitivity and response were not reduced in the mutant.

Rapid cell elongation has been shown to induce formation of transverse microtubules (Sambade *et al.*, 2012), suggesting that microtubule orientation into transverse arrays is a part of cellular response to rapid cell elongation, rather than being a licensing factor for commencing of anisotropic cell expansion. In the *ton2* mutant, the smaller number of transverse microtubules emerging from the transverse orthogonal walls under hormone-treatment conditions may be related to the inability of the *ton2* mutant to elongate rapidly. Indeed, cells of the *ton2* mutants are smaller in size, consistent with growth defects (Torres-Ruiz and Jurgens, 1994; Kirik *et al.*, 2012). Thus, reduced efficiency of microtubule reorientation into transverse arrays in the *ton2* mutant supports the hypothesis that rapid cell elongation drives reorientation of microtubules into transverse arrays.

In the *ton2* mutant the frequency of transverse microtubules appearing from the longitudinal cell sides was 4-times lower than in wild type. This inability to produce enough side microtubules may lead to slower reorientation rate in *ton2* mutants. The molecular mechanism generating these transverse microtubules is not known. It was suggested that transverse microtubules form at the anticlinal sidewalls of cells by a local increase in microtubule nucleation (Vineyard *et al.*, 2013). As branching nucleation is the only microtubule nucleation mode reduced in the *ton2* mutant, it is tempting to speculate that branching nucleation contributes to the formation of pioneering transverse microtubules. However, both in wild type and in the *ton2* mutant hormones increases the frequency of the transversal microtubules without an increase in the proportion of branching nucleation, casting a doubt on the role of branching nucleation as the primary generator of discordant microtubules. The other nucleation types, parallel and *de novo*, also did not increase at the periclinal cell surface during the hormone treatment. Consistent with previous observations, our data suggest that the mechanism generating discordant transverse microtubules is not active at cells’ periclinal surface, but rather is activated on the longitudinal orthogonal surface of the cell (Sambade *et al.*, 2012; Vineyard *et al.*, 2013). Our data, however, cannot exclude the possibility that microtubule nucleation is a part of the reorientation mechanism that locally up-regulates formation of new microtubules at longitudinal side walls of hypocotyl cells.

The results presented here show that despite a lower proportion of branching nucleation and reduced microtubule stability, most *ton2* mutant cells can still respond to the IAA/GA_4_ hormonal signal and initiate microtubule reorientation. Our data show that TON2 function is required for efficient reorientation by supporting the formation of transverse microtubules emerging at the anticlinal faces. It has been shown that microtubule density is 27% in the hypocotyl epidermal cells of the *ton2* mutant (Kirik *et al.*, 2012). The origin of the pioneering transverse microtubules during hormone-induced reorientation is unknown, but the density of pre-existing polymers is likely to play an important role in several possible mechanisms. For example, pre-existing microtubules can provide a surface for binding of the γ-TURC nucleation complexes and can form microtubule crossovers, inducing severing and formation of new microtubule plus ends. Transverse microtubules were shown to be present at the longitudinal anticlinal walls even in cells with longitudinally oriented arrays at the periclinal cell surface (Chan *et al.*, 2009). In this scenario, efficient generation and spreading of discordant transverse microtubules during the reorientation process will be dependent on the microtubule density and, consequently, *ton2* mutants will not be able to generate transverse microtubules at high frequency owing to their lower density of microtubules.

Discordant side microtubules appear from the longitudinal cell sides, suggesting that microtubule reorientation may be triggered by cell polarity signals. It is conceivable that the hormone auxin, which is known to polarize plant cells during development, may change cell polarity and growth direction of the hypocotyls cells when applied exogenously. Gibberellic acid is known to promote cell elongation (Shibaoka, 1974; Shani *et al.*, 2013). Therefore, directional cell expansion itself may provide a signal changing cell polarity and ultimately stimulating the formation of microtubules at the longitudinal anticlinal walls.

Addressing molecular mechanisms that generate transversal microtubules in response to hormones will be key to understanding how microtubule reorientation is triggered. To shed light on the mechanism triggering microtubule reorientation, it will be important to develop microscopy techniques that will allow resolution of individual microtubules at the anticlinal cell surface to test for changes in microtubule nucleation rates and local dynamic properties of microtubules during the reorientation process.

## Conclusions

Previous studies suggested that microtubule nucleation plays an important role in reorientation of microtubule arrays. We have found that nucleation efficiency, branching nucleation angles, and the ratios between branching, parallel, and *de-novo* nucleations at the periclinal cell surface were not changed during hormone-induced microtubule reorientation into transverse arrays, indicating that the microtubule reorientation mechanism does not involve modifications of microtubule nucleation at the periclinal cell surface. Although at a slower rate, microtubule arrays were able to reorient in the *ton2* mutant, which has reduced branching nucleation frequency and microtubule nucleation efficiency, suggesting that additional mechanisms, such as katanin-induced microtubule severing or stabilization of microtubules crossing cell edges are likely to be involved in locally up-regulating the production of transverse microtubules at the orthogonal sidewalls. However, a local up-regulation of microtubule nucleations at the orthogonal cell faces cannot be excluded and merits future investigation to elucidate mechanisms involved in microtubule array reorientation into transverse arrays.

## Supplementary data

Supplementary data are available at *JXB* online

Supplementary Fig. S1. Frequency of different nucleation types during 90min of hormone-induced microtubule reorientation.

Supplementary Fig. S2. Ratios of nucleation modes measured in experiments with reduced laser light exposure time.

Supplementary Data
