# Supplementary material for: Microtubule array reorientation in response to hormones does not involve changes in microtubule nucleation modes at the periclinal cell surface
Source: J Exp Bot. 2014 Aug 18;65(20):5867–75. doi: 10.1093/jxb/eru325 (PMC4203123; doi:10.1093/jxb/eru325)
Supplement: Supplementary Data [file supp_65_20_5867__index.html]

Microtubule array reorientation in response to hormones does not involve changes in microtubule nucleation modes at the periclinal cell surface — Microtubule array reorientation in response to hormones does not involve changes in microtubule nucleation modes at the periclinal cell surface — Supplementary Data 

# Microtubule array reorientation in response to hormones does not involve changes in microtubule nucleation modes at the periclinal cell surface

## Supplementary Data

Data files

**Files in this Data Supplement:**

- Supplementary Data - Supplementary Data
