# Supplementary material for: Microtubule array reorientation in response to hormones does not involve changes in microtubule nucleation modes at the periclinal cell surface
Source: J Exp Bot. 2014 Aug 18;65(20):5867–75. doi: 10.1093/jxb/eru325 (PMC4203123; doi:10.1093/jxb/eru325)
Supplement: Supplementary Data [file supp_eru325_jexbot125039_file001.pdf]

Supplementary Figure 1

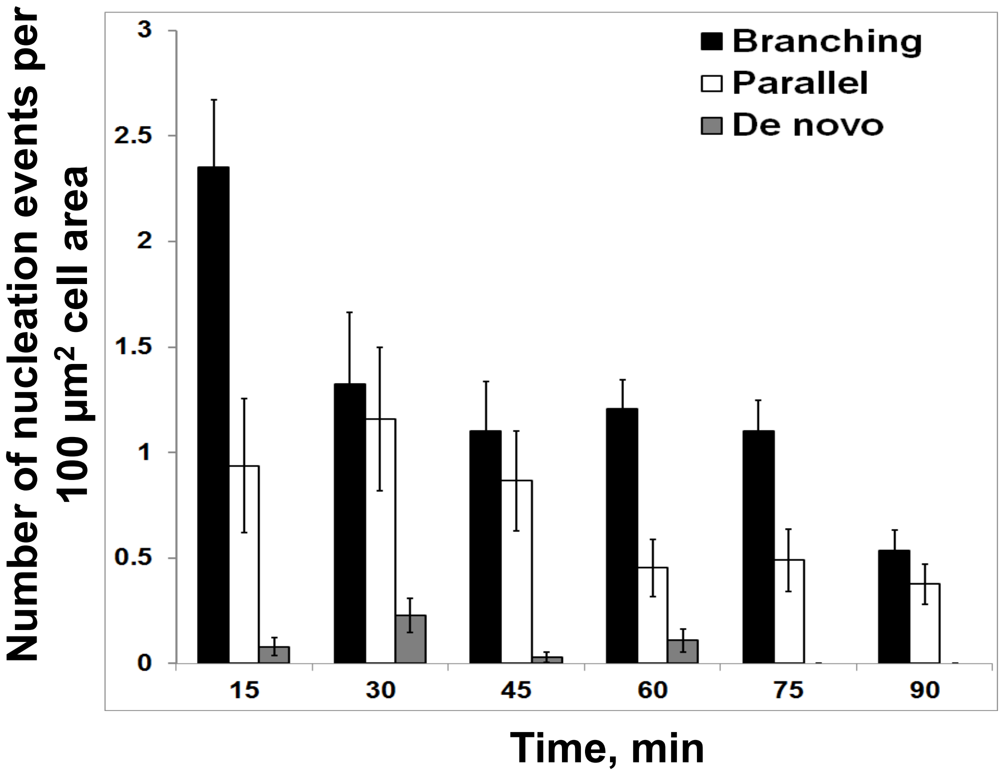

Supplementary Figure 1. Frequency of different nucleation types during 90 minutes of hormone-induced microtubule reorientation. Data were collected in 15 minute time bins over 90 minutes of hormone treatment. Number of cells n=4, error bars show standard error of mean.

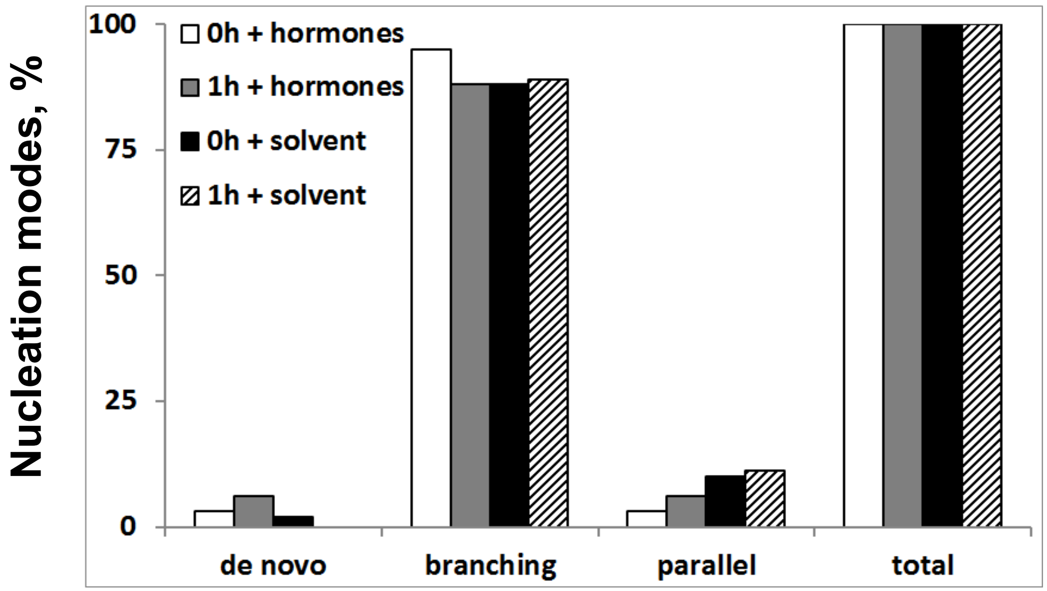

Supplementary Figure 2. Ratios of nucleation modes measured in experiments with reduced laser light exposure time. Time lapse images were taken in the first 5 minutes followed by another 5 minutes acquisition interval after 1 hour of hormone treatment.
